# Research progress on addictive features and reward circuit mechanisms in non-suicidal self-injury and the feasibility of precision neuromodulation

**DOI:** 10.3389/fpsyt.2026.1778566

**Published:** 2026-07-07

**Authors:** Jiao Li, Yan Zhang, Qiangli Dong

**Affiliations:** 1 The Second Hospital & Clinical Medical School, Lanzhou University, Lanzhou, China; 2 Department of Psychiatry, National Clinical Research Center for Mental Disorders, and National Center for Mental Disorders, The Second Xiangya Hospital of Central South University, Changsha, China; 3 China National Technology Institute on Mental Disorders, Hunan Technology Institute of Psychiatry, Changsha, China

**Keywords:** behavioral addiction, neuromodulation, non-suicidal self-injury, precision medicine, reward network

## Abstract

Non-Suicidal Self-Injury (NSSI) presents a significant public health challenge; however, its underlying neurobiological mechanisms remain insufficiently understood, limiting the development of targeted interventions. Emerging evidence suggests that NSSI exhibits core addictive features, such as compulsive urges and tolerance, which may be driven by dysfunctions in the brain’s reward circuitry. This review synthesizes current research on the neural overlaps between NSSI and addiction, specifically focusing on the dysregulation of the ventral striatum and prefrontal cortex. Based on this mechanistic framework, we propose the potential of Stanford Accelerated Intelligent Neuromodulation Therapy (SAINT)—a high-dose, functional connectivity-guided transcranial magnetic stimulation protocol—as a precision treatment for NSSI. By targeting specific reward network deficits, SAINT may offer a novel, rapid-acting therapeutic strategy for patients who do not respond to conventional pharmacological or psychological interventions.

## 1 Introduction

Non-Suicidal Self-Injury (NSSI) refers to the deliberate, direct destruction of body tissue without suicidal intent, with common manifestations including skin cutting, head banging, and burning (1). A 2024 systematic review and meta-analysis of global adolescent self-injurious behaviors revealed a 12-month prevalence of 16.2% and a lifetime prevalence of 21.7% for NSSI, indicating that approximately one in five adolescents worldwide has engaged in self-harm (2). The World Health Organization (WHO) identifies NSSI as the second leading contributor to disability among individuals aged 15–29 (3). Notably, NSSI demonstrates particularly high prevalence in populations with borderline personality disorder and depressive disorders. Beyond causing physical injury and social dysfunction, NSSI significantly elevates suicide risk, with empirical evidence confirming it as the strongest predictor of suicide attempt (4). Alarmingly, suicide has become the second most common cause of non-natural death among children and adolescents globally (5). These findings collectively underscore NSSI as an urgent public health challenge. Despite widespread recognition of its harms, the neurobiological mechanisms underlying NSSI remain incompletely elucidated, directly impeding the development of effective interventions.

Growing evidence suggests that NSSI exhibits salient addictive features, characterized by “recurrent, irresistible urges to engage in self-injury.” (6) Approximately 45.2% of individuals with NSSI meet criteria for addiction-like behavioral patterns (7). Mechanistically, adolescents with depression frequently employ self-injury as a maladaptive coping strategy to alleviate anxiety, depressive affect, and other negative emotional states (8). During NSSI episodes, individuals experience rapid emotional relief accompanied by excitement, euphoria, and substance-like “rewarding” sensations (9–11). This neurobehavioral cascade fuels intense cravings for self-injury, driving compulsive engagement despite awareness of its detrimental consequences. Over time, both the frequency and severity of NSSI escalate, mirroring the behavioral reinforcement patterns observed in addiction (9). Previous studies have proposed that dysfunction of the reward network may be a key neural mechanism in addiction (12–14). As a core network regulating motivation, emotion, and reward information processing, dysfunction of the reward network may lead to pleasure deficit, motivation deficit, and impaired emotion regulation, which may drive NSSI behaviors (14). The aim of this review is to integrate research advances related to NSSI addiction characteristics and reward networks, and based on this, to propose the potential of precision neuromodulation in the field of NSSI treatment.

A comprehensive literature search was conducted across the PubMed and Web of Science databases for relevant articles published from their inception up to May 2026. The primary search strategy utilized various combinations of the following core keywords: (“Non-Suicidal Self-Injury” OR “NSSI”) AND (“Reward Network” OR “Reward Circuitry” OR “Addiction”) AND (“Neuromodulation” OR “rTMS” OR “tDCS”). To capture the transdiagnostic nature of NSSI, secondary searches incorporated specific psychiatric comorbidities, including (“Major Depressive Disorder” OR “Depression” OR “Borderline Personality Disorder”). The inclusion criteria prioritized peer-reviewed original empirical studies, systematic reviews, and meta-analyses that investigated the neurobiological overlaps between NSSI, reward dysregulation, and emerging non-invasive brain stimulation interventions. Studies were excluded if they lacked a specific focus on the mechanistic pathways or clinical translation related to the core themes of this review, or if they were not published in peer-reviewed English-language journals.

## 2 Addictive features of NSSI

Recent studies suggest that NSSI may align with characteristics of behavioral addiction, manifesting as intense cravings for self-harm, uncontrolled repetition, and withdrawal-like responses. From the subjective perspective of individuals engaging in NSSI, they frequently associate their behavior with addiction, reflecting the profound difficulty they face in completely ceasing NSSI. A social media-based survey revealed that online communities of individuals with NSSI often use addiction-related terms or phrases (e.g., impulsivity, tolerance, relapse) to describe their experiences (15). This is concerning, as those who perceive NSSI as an addiction may face heightened risks of more frequent and severe self-injury, accidental harm, and increased suicidal ideation and behaviors (16). Research also highlights associations between NSSI and other addictive behaviors, such as substance use disorders and eating disorders, suggesting shared neurobiological mechanisms—particularly dysregulation in the dopamine-driven reward system (17).

Nevertheless, there remains controversy within the academic community regarding the complete conceptualization of NSSI as an addictive disorder. Regarding psychological mechanisms, studies propose that NSSI motivation predominantly stems from negative reinforcement (e.g., reducing distress), whereas substance use is often driven by positive reinforcement (e.g., seeking pleasure) (18). Additionally, cravings for substances appear more pervasive and persistent across contexts, whereas NSSI-related urges typically emerge only during negative emotional states (19). Furthermore, we wish to emphasize that a crucial distinction between NSSI and substance use disorders lies in the complex social interaction feedback loops inherent to NSSI. Research on substance addiction typically focuses more on the direct effects of the substance on the brain. However, when discussing NSSI, we cannot overlook its underlying “social dimension”—such as the motivation to seek social support or escape certain social responsibilities through this behavior. Some studies highlight that if we overlook this aspect and directly label NSSI as addictive behavior, it may increase patients’ stigma and overlook its nature as a comorbid symptom of emotional disorders such as depression (20).

In the neurobiological model of NSSI addiction, the dopaminergic system, opioidergic system, and hypothalamic-pituitary-adrenal (HPA) axis play pivotal roles. Chronic self-injurious behaviors induce the release of endogenous opioids such as beta-endorphins, providing short-term relief from psychological pain. However, prolonged engagement leads to opioid receptor tolerance and dependence, subsequently suppressing the synthesis and release of endogenous opioids through negative feedback mechanisms (10). Previous longitudinal studies have demonstrated that individuals with NSSI exhibit significantly lower beta-endorphin levels in cerebrospinal fluid compared to non-NSSI controls, further supporting this hypothesis (21). Additionally, NSSI may activate the mesolimbic dopaminergic pathway to generate transient positive emotional reinforcement, synergizing with the endogenous opioid system to establish a dual-reward mechanism (22). Notably, NSSI individuals display elevated basal cortisol levels and blunted acute stress responses, consistent with chronic stress adaptation patterns (23). The biochemical interaction between adrenocorticotropic hormone (ACTH) and beta-endorphins, both of which originate from the common precursor pro-opiomelanocortin (POMC), may further contribute to the development of addictive characteristics in NSSI (10). Furthermore, this opioid-driven addiction cycle is highly illustrative in the context of BPD. According to the Endogenous Opioid System theory of BPD, individuals with this disorder often exhibit abnormally low basal levels of endogenous opioids, which clinically manifests as chronic feelings of emptiness, emotional instability, and pain insensitivity (24). In this chronically opioid-depleted state, the physical tissue damage caused by NSSI triggers a massive compensatory release of endogenous opioids. Due to the compensatory hypersensitivity of opioid receptors, this sudden neurochemical surge provides immediate and profound relief from aversive inner tension. Consequently, for patients with comorbid BPD, NSSI transcends a simple coping mechanism; it becomes a powerful, neurochemically reinforced behavioral addiction driven by an urgent drive to “self-medicate” a deficient opioid system (25).

## 3 NSSI-related reward network mechanisms

The reward network mechanism in NSSI involves key brain regions such as the ventral striatum (VS), prefrontal cortex (PFC), cingulate gyrus (CG), amygdala (AMYG), hippocampus (HIP), and thalamus (THA). These regions collectively mediate various aspects of reward processing and regulate reward-related behaviors (26). Specifically, distinct subregions of PFC play opposing roles in this network: the orbitofrontal cortex (OFC) is primarily involved in encoding reward value and emotional processing, whereas the dorsolateral prefrontal cortex (DLPFC) is crucial for top-down cognitive control and the inhibition of impulsive behaviors (23). Among these, the VS—a critical component of the reward system—participates in action planning, risk decision-making, motivation, reinforcement, and reward perception. In research, the VS is often subdivided into the nucleus accumbens (NAcc), caudate nucleus (CAU), and putamen (PUT). Behaviorally, reward responses encompass three substructures: reward anticipation, initial response to reward, and reward satiation. Following NSSI engagement, individuals experience transient emotional relief through automatic negative reinforcement, alleviating preexisting emotional numbness and negative affect (11), while simultaneously obtaining pleasure or satisfaction via positive reinforcement. Over time, individuals may progressively escalate the frequency and severity of NSSI behaviors to elicit and sustain these reward responses (11). This escalation heightens the impulsivity and compulsivity of NSSI, ultimately transforming it into a habitual behavior—a phenomenon consistently described as the “addictive features” of NSSI (27).

In task-based fMRI studies employing symptom-provocation paradigms, individuals engaging in self-injurious behaviors exhibit enhanced activation in the OFC and CG compared to healthy controls (28, 29). Another study focusing on adolescents at risk for NSSI ideation (without actual NSSI behaviors) found that early adolescent NSSI ideation is significantly associated with bilateral PUT activation, and adolescents with NSSI ideation show higher average activation levels in the R-NAcc and R-PFC than controls (14). Regarding intrinsic network architecture, resting-state fMRI seed-based analysis further revealed stronger functional connectivity (FC) between the L-NAcc and the right lingual gyrus, R-NAcc, R-PUT and right angular gyrus in the NSSI group. Conversely, reduced FC was observed between the R-NAcc and left inferior cerebellar lobule, left CG and right angular gyrus, left CG and left middle temporal gyrus, and right CG with bilateral middle temporal gyri. Notably, this study also reported a positive correlation between FC strength between R-NAcc and the left inferior cerebellar lobe and NSSI addiction-like trait scores (12). Based on prior evidence, we hypothesize that dysfunction in the reward network may serve as a key neural mechanism underlying the addictive features of NSSI. According to the relief learning model, dopamine release in reward-related brain regions increases during pain or negative emotional relief, thereby reinforcing reward effects, whereas blocking dopamine release inhibits such effects (30). Additionally, NSSI promotes endorphin release, and these endogenous opioids generate a reward-driven “rush” or “high” post-injury (21), further supporting the role of reward circuitry in mediating NSSI addiction.

The dysfunction of reward-related brain regions suggests that NSSI is neurobiologically hypersensitive to reward stimuli, which likely drives repeated NSSI engagement in adolescents. However, the specific regulatory mechanisms underlying this phenomenon remain unclear. Current studies exhibit significant heterogeneity in findings, potentially due to small sample sizes, poor participant compliance, population heterogeneity, or unmeasured confounders. Alternatively, this variability might reflect undiscovered NSSI subtypes with distinct neural profiles. Future research should prioritize larger cohorts, rigorous control of confounders, and subtype stratification to clarify these mechanisms and generate robust conclusions.

A Critical Synthesis It is crucial to acknowledge that the reported neuroimaging findings in NSSI exhibit significant heterogeneity. A critical synthesis suggests that this variability is likely driven by differences in experimental paradigms and psychiatric comorbidities. First, paradigm specificity plays a key role. Studies utilizing Monetary Incentive Delay (MID) tasks consistently report blunted activation in the ventral striatum (VS) during reward anticipation, reflecting an “anhedonic” or “wanting” deficit (14). In contrast, paradigms involving pain offset or social exclusion often reveal hyper-activation in the amygdala and anterior insula, supporting a “negative reinforcement” model where relief from distress is disproportionately rewarding (29). Second, comorbidities such as Major Depressive Disorder (MDD) and Borderline Personality Disorder (BPD) confound these results. Striatal hypoactivation is most robust in NSSI samples with comorbid MDD, whereas limbic hyper-reactivity is more characteristic of BPD traits (4). Therefore, current evidence should be interpreted not as a uniform pattern, but as a context-dependent dysregulation of the fronto-striatal network. Specifically, neuroimaging studies correlating NSSI with BPD consistently demonstrate severe frontolimbic network dysregulation. fMRI investigations reveal that BPD individuals who engage in NSSI exhibit pronounced hyper-reactivity in limbic structures—particularly the amygdala and anterior insula—when exposed to negative emotional stimuli or interpersonal rejection cues (31).This limbic hyperactivity is coupled with a marked deficit in top-down cognitive control, mediated by reduced structural integrity and functional connectivity within prefrontal subregions, including the DLPFC and OFC (32). Within the framework of reward processing, this frontolimbic failure interacts directly with the fronto-striatal reward network: intense emotional distress in BPD triggers an urgent motivational drive for stabilization, thereby biasing the reward system to heavily over-encode the immediate “relief value” of pain offset via self-injury (32). Thus, neuroimaging evidence suggests that while depression-comorbid NSSI may be primarily characterized by anhedonic striatal hypoactivation, BPD-comorbid NSSI is predominantly driven by a frontolimbic emotional-regulatory failure.

## 4 Current status of NSSI intervention strategies

Current clinical guidelines recommend psychotherapeutic interventions, particularly Dialectical Behavior Therapy (DBT) and Cognitive Behavioral Therapy (CBT), as the first-line treatments for NSSI. These evidence-based approaches have demonstrated significant efficacy in reducing self-injury frequency and improving emotion regulation. However, they are not without limitations. In the field of psychotherapy, dialectical behavioral therapy, cognitive behavioral therapy and other commonly used psychotherapeutic methods have been proved to partially reduce the relapse rate of NSSI in adolescents, but their efficacy is obviously affected by the degree of patient trust and the level of executive functioning (33). A substantial subset of patients exhibits only partial response or experiences relapse following treatment cessation. Furthermore, these therapies are often resource-intensive and require long-term engagement (months to years) to achieve stabilization, which may not be sufficient for acute crises requiring rapid intervention. Consequently, there remains a critical need for adjunctive, rapid-acting biological treatments for patients who are resistant to standard care. Among pharmacological treatments, selective 5-hydroxytryptamine reuptake inhibitors (SSRIs) such as fluoxetine and sertraline, although widely used to alleviate co-morbid depressive symptoms, have limited effectiveness in targeting core behavioral interventions for NSSI, an randomized controlled trial (RCT) showed that there was no significant difference between SSRIs-treated and placebo groups in terms of the reduction rate of NSSI events, and that high-dose administration of the medication may increase adolescents’ risk of radicalization (34). Based on the previously mentioned relationship between the HPA axis and NSSI, some studies have also innovatively suggested that opioid receptor antagonists such as naltrexone or adrenocorticotropic hormone releasing factor type 1 (CRF1) receptor antagonists could be used as a potential modality for the precision pharmacological treatment of NSSI, but further clinical validation is still needed (10). Current physical therapy does not have a strategy to target NSSI, and there is a lack of research in this area, with the majority of studies reporting partial reduction of NSSI behaviors by treating co-morbid mood disorders to achieve negative mood relief. Existing intervention strategies mostly lack precise targeting of neural circuits for NSSI, and the need for more precise and effective physical therapy for NSSI as a separate symptom is clinically crucial for patients.

## 5 The potential of targeted rTMS in NSSI treatment

Repetitive Transcranial Magnetic Stimulation (rTMS), a non-invasive neuromodulation technique based on the principle of electromagnetic induction, has been widely used in clinical practice to modulate brain network activity by inducing cortical currents through magnetic fields (35). Magnetic stimulation crosses the barrier of the scalp, skull and meninges and is converted into electrical stimulation on the surface of the cerebral cortex, modulating the activity of cortical neurons. The frequency at which the magnetic field stimulus is delivered to the brain determines whether the underlying brain structures are stimulated or inhibited (35). rTMS with a frequency of ≤1Hz is called low-frequency stimulation, which has an inhibitory effect; high-frequency stimulation ≥5Hz induces an excitatory effect in the brain. rTMS, as a non-invasive and safe neuromodulation technique, currently has some clinical efficacy in depression disorders, can reduce patients’ suicidal thoughts and self-injurious behaviors to a certain extent, and is more effective when used in conjunction with medication (36).

Crucially, the therapeutic potential of rTMS extends beyond mood disorders to specifically target the neural circuitry of addiction and compulsivity. Emerging evidence indicates that high-frequency rTMS applied to the prefrontal cortex can effectively restore top-down inhibitory control over dysregulated reward networks, thereby reducing craving and impulsive behaviors (37). Notably, the FDA has already cleared TMS protocols for OCD and smoking cessation, validating its efficacy in modifying rigid, habitual behaviors (38, 39). Pivotal multicenter studies have demonstrated that stimulating the prefrontal cortex and insula significantly reduces craving and relapse rates in nicotine addiction by modulating the excitability of the hypoactive executive control network. Similarly, in substance use disorders, rTMS has been shown to alleviate drug craving and improve decision-making by rectifying the imbalance between the prefrontal “stop” system and the striatal “go” system (40). These findings provide a compelling rationale for repurposing neuromodulation strategies to treat NSSI, which shares core phenomenological features—such as intense urges and a loss of control—with these established indications.

Given the tight transdiagnostic relationship between BPD and self-injurious behavior, exploring the clinical utility of non-invasive brain stimulation (NIBS) in BPD populations provides critical insights into targeting NSSI. Accumulating evidence from systematic reviews and RCTs confirms that rTMS and tDCS can significantly attenuate core BPD features, such as impulsivity and emotion dysregulation (41–45).Crucially, several of these trials have documented direct secondary reductions in NSSI behaviors. For instance, a pilot study by Reinsberg et al. (46) demonstrated that applying inhibitory 1 Hz rTMS over the OFC—a region we hypothesized to over-encode reward valuation—not only alleviated depressive symptoms but also yielded significant reductions in NSSI frequency among patients with comorbid BPD. Similarly, high-frequency rTMS targeting the dmPFC or prefrontal-limbic networks has led to substantial clinical improvements in borderline symptomatology alongside marked declines in self-harming behaviors (47, 48). Case report further substantiate that high-frequency TMS protocols can successfully suppress acute self-harm urges by restoring prefrontal regulatory capacity (49). Finally, in highly unstable patients presenting with severe suicidality and treatment-resistant depression, a feasibility trial combining Magnetic Seizure Therapy with DBT demonstrated concurrent reductions in NSSI (50). Taken together, these intervention outcomes provide a compelling, clinically validated rationale that modulating frontolimbic and fronto-striatal nodes can successfully curb compulsive self-injury.

The stimulation mode intermittent theta pulse stimulation (iTBS) enhances synaptic plasticity by simulating θ rhythms (50Hz intra-cluster frequency/5Hz inter-cluster frequency), which has a faster onset of action and a higher dosage than traditional rTMS. In recent years, the Stanford research team has made innovative modifications based on the iTBS framework (51): 1) treating patients multiple times per day at optimal intervals (10 times per day for 5 consecutive days), 2) applying higher pulse stimulation (1,800 pulses per session at 50-minute intervals), and 3) pinpointing targeting of brain regions via resting-state functional MRI. This method, known as Stanford Accelerated Intelligent Neuromodulation Therapy (SAINT), is mainly used overseas for the treatment of refractory depressive disorders, and data show that 90.5% of subjects with depressive disorders who received this method achieved clinical remission of their symptoms, which proves that its efficacy is significantly better than that of the current general rTMS treatment, and its safety monitoring shows that it did not cause serious adverse events or cognitive impairment. The original SAINT program targeted the neural subregion of the left dorsolateral prefrontal cortex that is most negatively correlated with the function of the subglottic anterior cingulate cortex. Based on the hypothesis of reward network dysregulation in NSSI and the safety and efficacy of SAINT, we speculate that if future research can target the addictive characteristics of NSSI and utilize SAINT to precisely modulate the reward-related brain regions, we will be able to achieve clinical relief of the symptoms of NSSI with SAINT. By modulating the core targets related to reward brain regions, it is possible to significantly reduce the frequency of NSSI behaviors.

Specifically, regarding potential stimulation targets, based on the evidence summarized above, the OFC and VS emerge as critical candidate nodes within this dysregulated network. First, the OFC serves as a core region involving the representation, evaluation, and modulation of rewards. It encodes the value of different reward types on a common scale and modulates emotional responses accordingly, thereby directly influencing decision-making. fMRI evidence demonstrating OFC hyperactivity during NSSI urges may reflect an “over-encoding” of the “relief value” of self-injury, erroneously marking it as a high-priority reward option (52). Therefore, it is reasonable to hypothesize that an inhibitory protocol targeting the OFC could theoretically attenuate this pathological value assessment, thereby dampening the intense craving for NSSI. On the other hand, the VS acts as the “motor engine” of the reward circuitry, primarily responsible for encoding incentive salience and generating reward anticipation. As noted in the neuroimaging section, NSSI patients not only exhibit aberrant VS activation during reward anticipation tasks. but, crucially, resting-state analyses indicate that VS-cortical connectivity strength is positively correlated with addictive traits in NSSI (12). This suggests a central role for the VS in maintaining addictive self-injurious behaviors. While the anatomical depth of the VS limits the feasibility of direct TMS stimulation, this does not preclude it as a therapeutic target. Instead, “indirect targeting” offers a viable alternative strategy. Recent translational research demonstrates that the VS can be effectively modulated trans-synaptically via specific subregions of the L-DLPFC (53). By stimulating the specific superficial frontal node that exhibits maximal functional connectivity with the VS, this approach holds the promise of remotely remodeling this deep reward hub, thereby ameliorating motivational deficits and restoring impulse control.

The application of SAINT to the NSSI population necessitates a discussion of the associated safety concerns. For instance, a primary theoretical concern is whether high-dose, high-frequency stimulation might inadvertently exacerbate physiological arousal or impulsivity in individuals with pre-existing emotion dysregulation, and for adolescent patients, is it possible that this could affect their neurodevelopment? Given the current absence of direct safety data for SAINT in isolated NSSI populations, safety profiles must be cautiously extrapolated from related high-risk cohorts. Notably, pivotal trials of SAINT in treatment-resistant depression included patients with acute suicidal ideation and reported no side effects related to agitation or increased self-harm. Although these findings provide preliminary support for the protocol’s tolerability, they do not guarantee safety in NSSI-specific phenotypes. Therefore, future pilot studies require rigorous risk mitigation strategies, including the exclusion of seizure history, continuous monitoring in inpatient settings during the intensive stimulation period, and pre-defined “stopping rules” for emergent agitation. These safety measures are essential for exploring the potential of rapid-acting neuromodulation while adhering to ethical standards.

## 6 Conclusion

Current research suggests that NSSI has a widely recognized harmfulness as a global public health problem, that it has addictive characteristics and is closely associated with abnormal reward network functioning, but specific regulatory mechanisms need to be further explored in the future, and that existing interventions related to NSSI face clinical bottlenecks of insufficient targeting and poor efficacy at the level of technical application. Future studies should employ longitudinal analyses that follow early adolescence to early adulthood to better elucidate how changes in reward sensitivity over time contribute to the etiology and maintenance of NSSI and other suicide-related behaviors. Identification of reward targets associated with NSSI addiction traits and validation of targeted interventions should also be conducted to develop more precise and effective physical treatments for NSSI as a separate symptom, rather than just treating co-morbid mood disorders.
